# A New Corrosion Sensor to Determine the Start and Development of Embedded Rebar Corrosion Process at Coastal Concrete

**DOI:** 10.3390/s131013258

**Published:** 2013-09-30

**Authors:** Chen Xu, Zhiyuan Li, Weiliang Jin

**Affiliations:** Institute of Structural Engineering, Zhejiang University, Hangzhou 310058, China; E-Mail: zjulzy@zju.edu.cn

**Keywords:** corrosion sensor, cement mortar resistance, corrosion rate, reference electrode, EIS

## Abstract

The corrosion of reinforcements induced by chloride has resulted to be one of the most frequent causes of their premature damage. Most corrosion sensors were designed to monitor corrosion state in concrete, such as Anode-Ladder-System and Corrowatch System, which are widely used to monitor chloride ingress in marine concrete. However, the monitoring principle of these corrosion sensors is based on the macro-cell test method, so erroneous information may be obtained, especially from concrete under drying or saturated conditions due to concrete resistance taking control in macro-cell corrosion. In this paper, a fast weak polarization method to test corrosion state of reinforcements based on electrochemical polarization dynamics was proposed. Furthermore, a new corrosion sensor for monitoring the corrosion state of concrete cover was developed based on the proposed test method. The sensor was tested in cement mortar, with dry-wet cycle tests to accelerate the chloride ingress rate. The results show that the corrosion sensor can effectively monitor chloride penetration into concrete with little influence of the relative humidity in the concrete. With a reasonable corrosion sensor electrode arrangement, it seems the Ohm-drop effect measured by EIS can be ignored, which makes the tested electrochemical parameters more accurate.

## 1. Introduction

In recent years, chloride-induced corrosion of structural steel has caused serious damage to concrete structures all over the World. A large number of harbor bridges, dams, docks and harbor structures have been damaged by chloride penetrating from the surrounding environment, especially in tidal zones and coastal areas [1–4]. Currently, no design theories based on reliability has been widely accepted around the World to determine whether an important infrastructure can be in service for 100 years or above. In developed countries, “durability redesign” is often used according to the key parameters of structural *in-situ* durability provided by continuous dynamics on condition that information feedback concerning the key parameters can be obtained dynamically [4–8]. With embedded sensors, accurate information about the *in-situ* durability parameters of coastal concrete structures, such as corrosion current density, concrete resistivity, temperature and concentration of chloride ions, can be provided to determine corrosion conditions of concrete and offer definite corrosion front diagnosis information. Based on this information, an early warning on structural durability is expected to be given to guide the durability redesign or policy-making of response plans, prepare maintenance strategies and durable measures as early as possible and supervise the effectiveness of the corrosion control measures and maintenance plans, so that the best time for maintenance and repair won't be missed [9–12].

**Figure 1.**
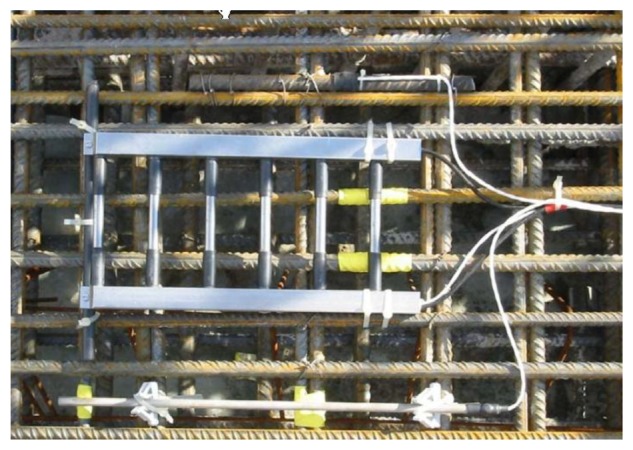
Anode-Ladder-System sensor.

**Figure 2.**
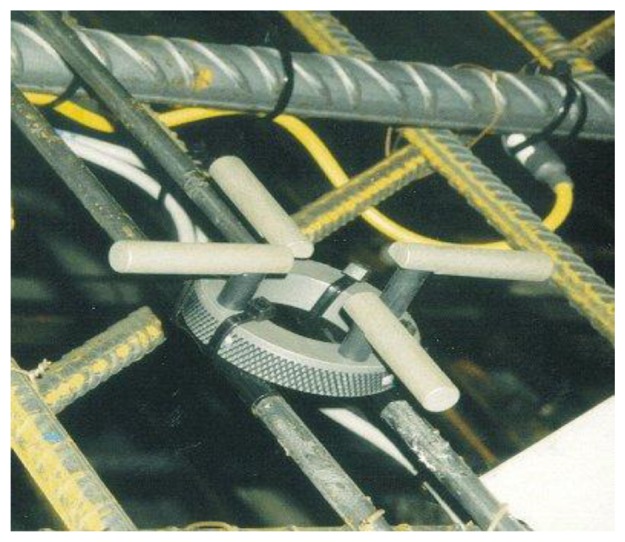
Nagel-System sensor.

**Figure 3.**
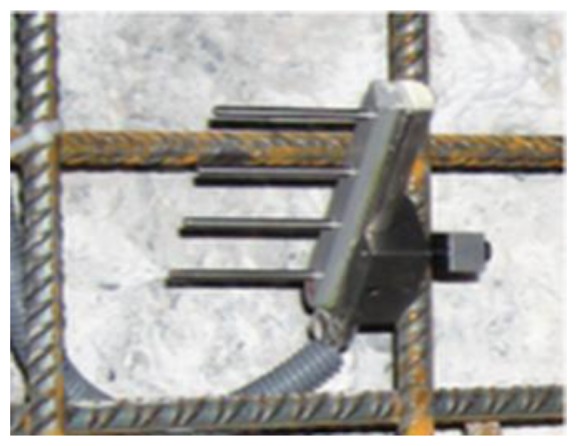
Senscore sensor.

In late 1980s, Europe started to develop corrosion monitoring systems, in which the Anode-Ladder-System (Figure 1) developed by Germany's S+R SensorTech and the Nagel-System (Figure 2) developed by Denmark's FORCE Technology are applied in many large concrete constructions in Europe and Africa [13,14]. Both of them install the sensors inside the structure and give an early warning on the corrosion time of reinforcements according to the depassivation corrosion conditions of anodes at different heights. Based on the German trapezoid anode inspection principles, the ROCKTEST company in Canada developed the SENSCORE system (Figure 3) in recent years. However, as a new market entry, this system hasn't been used widely in construction yet.

**Figure 4.**
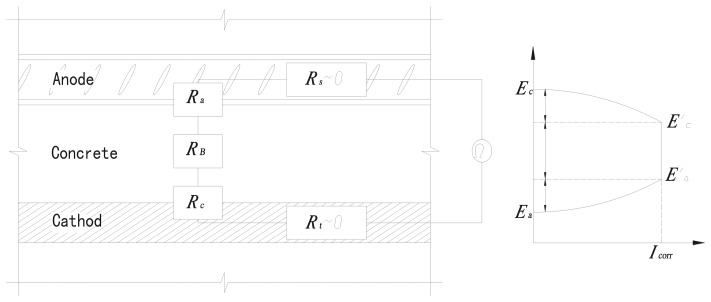
Equivalent circuit for corrosion closed circuit.

In the three corrosion monitoring systems mentioned above, the depassivation of anodes at different depths is determined according to the macrocell corrosion principle in electrochemistry. Specifically, macrocell corrosion usually appears if the distance between passivation area and active area is relatively large during the corrosion of anodes. The corresponding equivalent circuit is shown in Figure 4. If the resistance *R*_t_ of a reinforcement body in the passivation area (cathode) and the resistance *R*_s_ of a reinforcement body in the active area (anode) are omitted, the corrosion current follows Ohm's law in a closed circuit:

(1) $$I_{\text{corr}}=(E_{\text{a}}-E_{\text{c}})/(R_{\text{B}}+R_{\text{a}}+R_{\text{c}})$$

where *I*_corr_ is corrosion current; *E*_a_ and *E*_c_ are the equilibrium potentials on the anode and cathode; *R*_B_ is the resistance of the concrete; *R*_a_ and *R*_c_ are the reaction resistances on the anode and cathode.

According to the macrocell corrosion principle, when the sensor's anode is changed to an active area after depassivation, the equilibrium potential *E*_a_ will decrease substantially while the equilibrium potential *E*_c_ in the passivation area of cathode are almost maintained unchanged, resulting in a great increase in the potential difference between cathode and anode. If the impact of *R*_B_, *R*_a_ and *R*_c_ are not taken into consideration, the corrosion current *I*_corr_ (represented as corrosion macro current) will also increase largely.

However, a lot of research shows that when the relative humidity in concrete is at a general or lower level, microcell corrosion dominates due the large resistivity of concrete; only when the relative humidity in concrete is very large (more than 95%) and the concrete resistance *R*_B_ is very small, macrocell corrosion can become dominant, but excessive internal humidity will cause the collection of electrons on the surface of the anode, resulting in a distinct negative shift of equilibrium potential *E*_a_. Even when the anode is passivated, the measured macro current will increase greatly, reflecting an illusion of depassivation [15–17]. Therefore, macro current measurements are only applicable for general humidity conditions. Meanwhile, the distance between cathode and anode must be very small, otherwise the measured macro current will be smaller due to the impact of concrete resistance, making it unsure for judging the corrosion status of reinforcement. Especially when the corrosion monitoring is performed on concrete under the water where the sensor anode in concrete is in high humidity and oxygen deficit, the three kinds of sensors mentioned are not applicable in this case.

According to the analysis above, this paper designs a new corrosion sensor based on a three-electrode electrochemical test architecture. It also proposes an anodic polarization current method to determine the corrosion of reinforcement based on electrochemical polarization and quantifies the relation between relevant evaluation index and corrosion current. Through the finite element analysis of the polarized current field and the electrochemical testing and analysis of monitoring points, the sensor's performance is evaluated.

## 2. Experimental

### 2.1. Materials

P·O 42.5 cement from Qianchao Cement Co. Ltd. (Hangzhou, China) was used for all experiments in this study. River sand with a fineness modulus of 2.4 was used as a fine aggregate. Q235 steel was used for working electrodes of the corrosion sensor. Q235 steel is widely used in China in civil engineering and especially in coastal construction projects.

### 2.2. Sensor Arrangements

**Figure 5.**
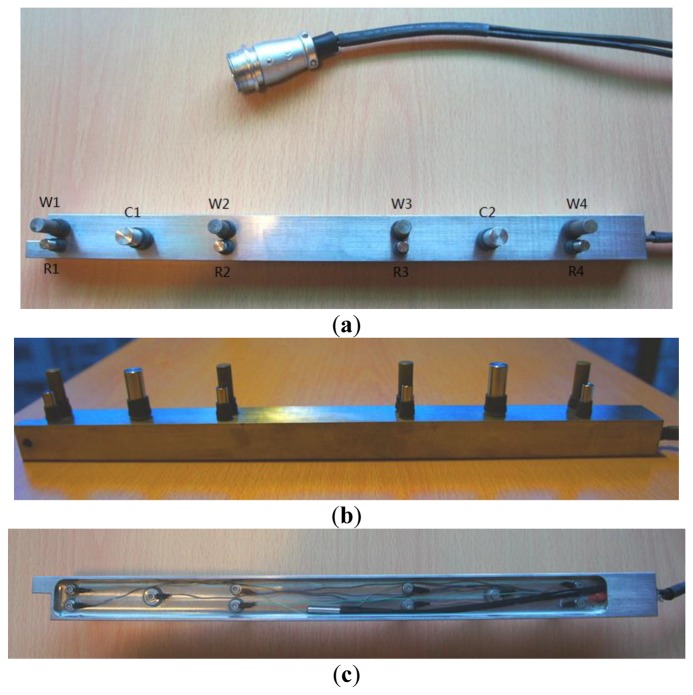
The geometrical sizes of the sensor.

In the anode ladder-type sensor, as shown in Figure 1, the larger diameter aggregate can easily be placed at the upper ladder of the anode and hardly sink during concreting, resulting in uneven mixing. Therefore, a new sensor in Figure 5 is designed. The sensor is arranged on one side to reduce the probability that coarse aggregate is laid up. In Figure 5a, W1, W2, W3 and W4 are Q235 steel bars with a diameter of 8 mm are used to monitor the corrosion fronts as working electrodes. R1, R2, R3 and R4 are titanium bars with a diameter of 6 mm used as reference electrodes. C1 and C2 are stainless steel bars with a diameter of 10 mm used as counter electrodes. To reduce the influence of the ohmic drop of concrete, the distance between reference electrode and working electrode is only 3 mm. During measurements, the counter electrode C1 is used to polarize working electrodes W1 and W2 and the counter electrode C2 is used to polarize working electrodes W3 and W4.

In Figure 5b, the working electrode shows a 2.5 cm long base. To prevent crevice corrosion, the bottom is sealed with heat shrinkable tube. The exposed length of working electrode is about 2 cm. In order to ensure the long-term stable working of the electrode connection in concrete, the base line is sealed with epoxy resin on the back, as shown in Figure 5c.

**Figure 6.**
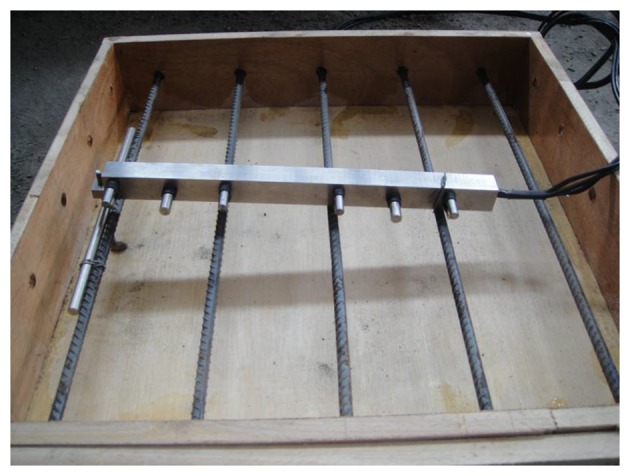
Sensor installation.

A concrete block with a size of 50 cm × 50 cm × 10 cm was prepared, using the concrete mixture proportions listed in Table 1. A 12 mm diameter piece of reinforcement was placed at 10 cm intervals in one direction of the block, and the thickness of protective layer was set at 4 cm. The sensor was put on the upper reinforcement, as shown in Figure 6. Then the sensor dip angle was changed by adjusting the screw length on one side of the sensor. The thickness of the protective layer at adjusted monitoring points is 0.9 cm, 1.6 cm, 2.3 cm and 3 cm, as shown in Figure 6.

### 2.3. Experimental Process

The concrete block was cured for 28 days at an ambient temperature of 20 ± 1 °C and a relative humidity (RH) of 95%. Afterwards, the block is placed indoors for one month of air drying, and then placed in a closed box with 5% NaCl solution. By dry-wet circulation with soaking in NaCl solution for 5 days and air drying for 2 days, chloride can ingress into the concrete more faster.

### 2.4. Measurement Theory and Procedure

#### 2.4.1. Anodic Polarization Current (APC)

In general, the cathode reaction includes both electrochemical polarization and concentration polarization, *i.e.*, the hybrid control of cathodic process. Equation (2) is the polarization curve equation when the corroding metal electrode is in weak polarization region:

(2) $$I=I_{\text{corr}}\;\left\{\exp\;\left(\frac{\Delta E}{\beta_{a}}\right)-\frac{\exp\;\left(-\frac{\Delta E}{\beta_{c}}\right)}{1-\frac{I_{\text{corr}}}{I_{L}}\left[1-\exp\;\left(-\frac{\Delta E}{\beta_{c}}\right)\right]}\right\}$$

where *I* is the polarization current; *I_corr_* is the corrosion current; *ΔE* = *E* − *E_corr_* is the polarization value of corroding metal electrode; *β_a_*, *β_c_* are the Tafel slopes of anode and cathode; *I_L_* is the limit diffusion current of cathodic reaction. When *I_L_* » *I*_corr_, cathodic reaction is controlled by the electrochemical reaction process, *i.e.*, the concentration polarization of the cathodic reaction in the corrosion can be ignored, which is called the “corrosion system controlled by activation polarization”, so Equation (2) is changed to a common polarization curve equation in a weak polarization region:

(3) $$I=I_{\text{corr}\;}\left\{\exp\;\left(\frac{\Delta E}{\beta_{a}}\right)-\exp\;\left(\frac{-\Delta E}{\beta_{c}}\right)\right\}$$

when the measured electrode is in the passivation state, the resistance in the anodic process is quite large, *i.e.*, *β_a_* tends to infinity and *I_corr_* tends to zero. Thus, the Equation (2) is changed to:

(4) $$I=I_{\text{corr}}\;\left\{1-\exp\;\left(\frac{-\Delta E}{\beta_{c}}\right)\right\}$$

If the polarization overpotential Δ*E* align is kept constant, the anodic polarization current will increase distinctly as *β_a_* reduces sharply and *I_corr_* increases during the depassivation of the reinforcement. In this case, despite of a possible slight increase in *β_c_*, the anodic polarization current rise still cannot be reversed due to the big drop of *β_a_*. Therefore, the sharp increase of anodic polarization current can be used as a criterion of the reinforcement depassivation.

The three-electrode method is used for anodic polarization test at the sensors' monitoring point, in which Δ*E* is set to 50 mv and the scan rate to 0.15 mV/s. The polarization starts from the equilibrium potential and ends upon reaching the relative equilibrium potential +50 mV, and then anodic polarization current *I_APC_* at the end moment is recorded.

#### 2.4.2. Electrochemical Impedance Spectroscopy (EIS)

**Figure 7.**
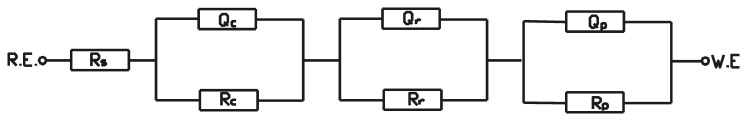
Equivalent circuit of the corrosion sensor embedded in the cement mortar.

EIS tests were conducted at the rest potential in the frequency range of 10^−3^ Hz to 10^6^ Hz using signal amplitude of 5 mV. The real part (Z_re_) and the imaginary part (Z_im_) of the sensor cell impedance were recorded [18]. It is worth noting that the charging of a discontinuous, inhomogeneous interface between the cement mortar and the steel anode leads to a Constant Phase-angle Element (CPE)-like response. A response of this type in the steel anode-mortar system is therefore to be expected, due to both the lack of surface homogeneity in the reinforcements and the eminently heterogeneous nature of mortar [19]. Consequently, the modified circuit shown in Figure 7 was used to quantitatively interpret the electrical signal response of the steel anode-mortar system.

The elements *Q*_c_ represents the capacitance of the cement material between the reference electrode and working electrode. *Q_r_* represents the capacitance of rust layer after corrosion. *Q*_p_ represents the interfacial capacitance of the working electrode. *R*_c_、*R*_p_ and *R_c_* represent the cement material resistances between reference electrode the charge transfer resistance and rust layer resistance, respectively. *R*s represents the solution resistance. The symbol *Q* usually denotes a CPE element, and the impedance of *Q* can be given as follows:

(5) $$Z=\frac{1}{Y_{0}}\times {\left(J\omega \right)}^{-\text{n}}$$

where n is a constant, *Y*_0_ (in Ω^−1^·cm^−2^·s^−n^) is a parameter derived from the capacitance *C* (in F), and *w* is the frequency.

#### 2.4.5. Linear Polarization Method

The polarization resistance of the electrodes was determined in each of the cement mortar samples. The potential was swept at a scan rate of 0.2 mV/s, from −20 to 20 mV referenced against the free corrosion potential of the steel anode. Measurement configurations with three electrodes were used, with each steel anode acting as a working electrode, the stainless steel bars acting as a counter electrode and the titanium bars acting as a reference electrode. The polarization resistance *R*_p_ (in Ω) can be deduced from the response Δ*I* (in *u*A·cm^−2^) of the steel anodes to a small amplitude step of potential Δ*E* (in mV):

(6) $$R_{P}=\Delta E/\Delta I_{\left(\Delta E\to 0\right)}$$

The corrosion potential was set at 26 mV for active corrosion and 52 mV for passive state. Then, the corrosion rate was calculated by Equation (7):

(7) $$I_{\text{corr}}=\frac{B}{R_{P}}$$

where *I*_corr_ is the corrosion rate, *B* is the constant corrosion potential and *R*_P_ is the polarization resistance calculated by Equation (6).

## 3. Results and Discussion

### 3.1. Impact of the Polarization Current Field

**Figure 8.**
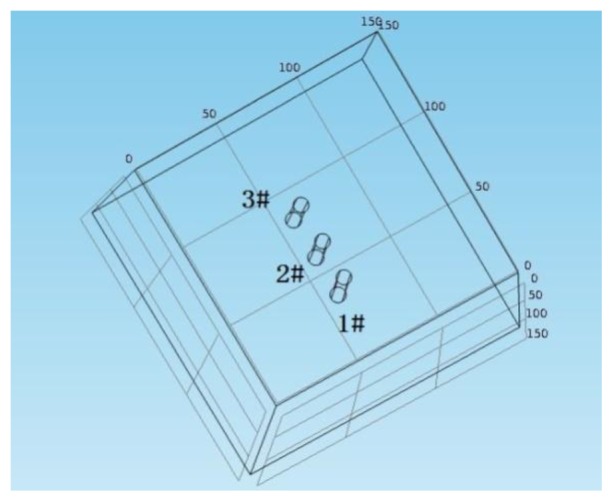
Finite element model diagram.

**Figure 9.**
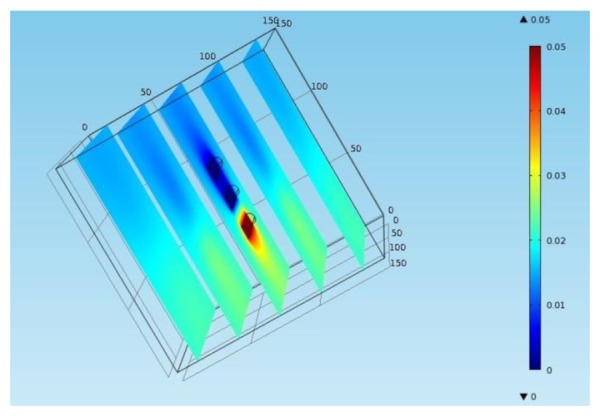
Electric potential contour.

The finite element software COMSOL Multiphysics is used to establish a finite element model as shown in Figure 8. The size of concrete block is 150 mm × 150 mm × 150 mm and three iron bars with a diameter of 8 mm and a length of 50 mm are placed at the center of block and spaced at a clear distance of 14 mm. The conductivity of concrete is 0.005 S/m and the conductivity of the iron bars is 1.12 e7 S/m. The initial voltage in the block is 0 V. 50 mV voltage is applied on the iron bar #1 and #2 to simulate the polarization impact of iron bar #2 (counter electrode) on iron bar #1 (working electrode). Figure 9 is the potential sectional drawing from finite element simulation. The figure shows that when the polarization current polarizes the iron bar #1 to 50 mV, the anodic polarization current field will not have any polarization effects on the iron bar #3 (working electrode). In contrast with the electrode distribution of sensors, after the counter electrode C1 polarize the working electrode W1, the polarization test can be immediately applied to the working electrode W2 without considering the relaxation effect of polarization current field on the working electrode W2.

### 3.2. Characteristics of the Anodic Polarization Curve During Curing

Equilibrium potential and anodic polarization current were tested at each point monitored by sensors after a ten-day block curing. Table 2 shows the equilibrium potential at each monitoring point. To evaluate the potential of titanium electrode, a contrast test was applied with a saturated calomel electrode. Table 2 shows that the potential decreases as the embedded depth increases, and changes at a slower pace at the first three monitoring points but falls sharply at the monitoring point W4 (mainly related to oxygen supply). During curing, the humidity inside the block is high. The oxygen deficiency at each monitoring point leads to the accumulation of negatron and the negative shift of equilibrium potential. As the embedded depth increases and oxygen deficiency worsens, the negative shift of potential also becomes more obvious. In addition, the potential of saturated calomel electrode shows the equilibrium potential at each monitoring point is apparently lower than the corrosion potential threshold (−280 mV) [20]. If it is reflected on the macro current, larger current fluctuations will appear. This also explains why a significant increase of macro current will occur in anodic ladder-type sensor at the curing stage.

**Figure 10.**
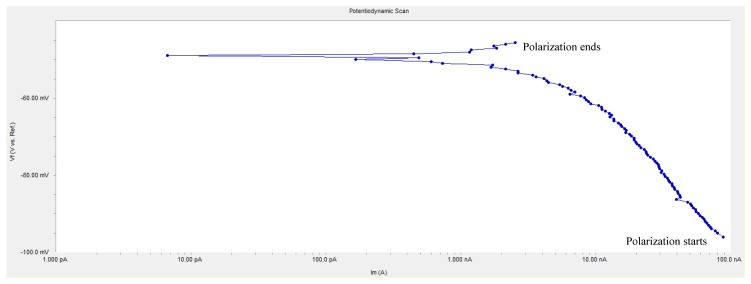
Anodic polarization curve of monitoring point W2.

An anodic polarization curve test was applied at each monitoring point, and the anodic polarization current I_APC_ is shown in Table 3. In the table, the value at some individual monitoring point is negative, which conflicts with polarization principle. Figure 10 shows the anodic polarization curve of the measured monitoring point W2. The curve has the typical cathodic polarization characteristics, but the start position of polarization is exchanged with the end position. According to the principle of polarization, the initial polarization current should be near zero, but it starts from −90 nA and ends at 2.529 nA in Figure 10. This is because the concrete in the curing period has a great internal humidity and the accumulation of negatron leads to a negative shift of equilibrium potential, *i.e.*, the measured equilibrium potential at monitoring points is not the real equilibrium potential; if anodic polarization starts with this potential, *i.e.*, the initial polarization potential is lower than real equilibrium potential, the cathodic polarization curve as shown in Figure 10 will appear. Besides, in Figure 10, the polarization current is at the nA level and the absolute value of the changing polarization current is only 92.529 nA, showing that the monitoring point is in the passivation state. It can be seen that if the passivated reinforcement in concrete is under high humidity and oxygen deficit, the equilibrium potential will still reduce to a value below the corrosion threshold and influence the judgment of the corrosion state of the reinforcements, while the value of anodic polarization current may be negative or very small, but it will not affect the judgment of the corrosion state.

**Figure 11.**
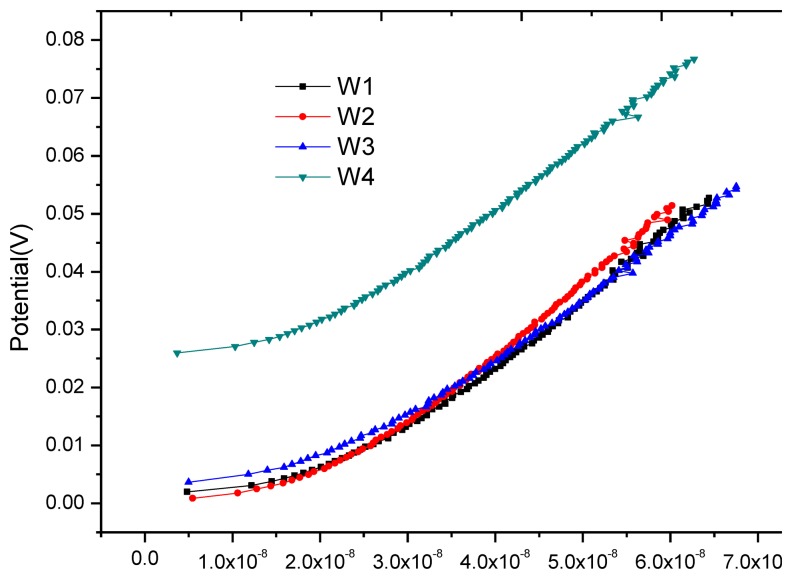
Anodic polarization curve for each monitoring point after drying for one month.

Having been cured for 28 days, the block was placed indoors for one month of air drying. Then the anodic polarization current test was applied at each monitoring point. Figure 11 shows the anodic polarization curve at each monitoring point. It can be seen that the equilibrium potentials at monitoring points W1, W2 and W3 are close and the equilibrium potential at W4 is higher, but the changing trend of all polarization curves is consistent. Their anodic polarization current *I_APC_* are close at the end point of polarization (see Table 4), and far less than the critical corrosion anodic polarization current *I_cr_* = *i_cr_* × S = 0.2 μA/cm^2^ × 3.14 × 0.8cm × 1.5cm = 0.754 μA, showing that all monitoring points are in stable passivation. Here *i_cr_* is the critical corrosion anodic polarization current density determined by Equation (9) in Section 3.4; A is the exposure area of monitoring point W1.

### 3.3. Characteristics of the Anodic Polarization Curve During the Test

**Figure 12.**
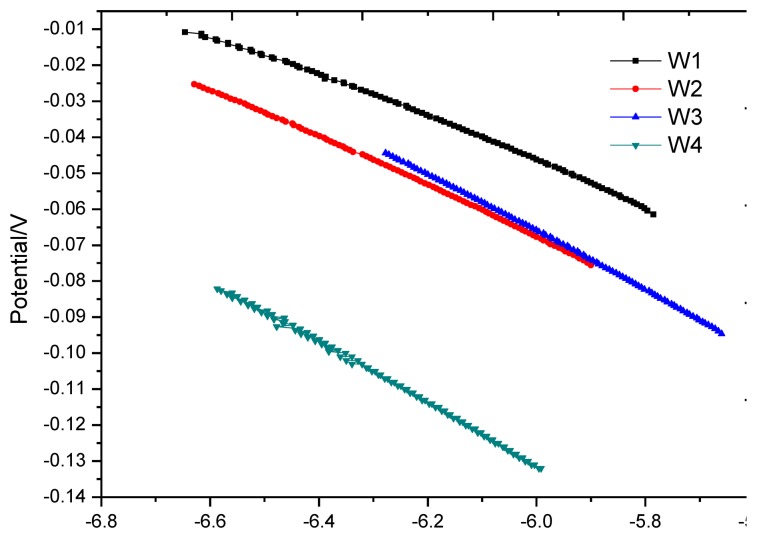
Anodic polarization curve for each monitoring point after first wetting cycle.

At the end of the soaking process in the first dry-wet circulation, the anodic polarization curve test should be immediately applied to the monitoring points. The test results are shown in Figure 12. In the figure, the anodic polarization curves at the monitoring points approximate a straight line and are close in slope. The curve shape is similar to the cathodic polarization curve in the strong polarization area [18]. This is because each monitoring point is in oxygen deficit at the end of soaking and the measured equilibrium potential has been moved to the cathodic strong polarization area. As the embedded depth of the monitoring points increases, the degree of oxygen deficit increases, and the negative shift of equilibrium potential also increases. The shape of polarization curves at point W3 and W4 is more close to a straight line than those at point W1 and W2.

**Figure 13.**
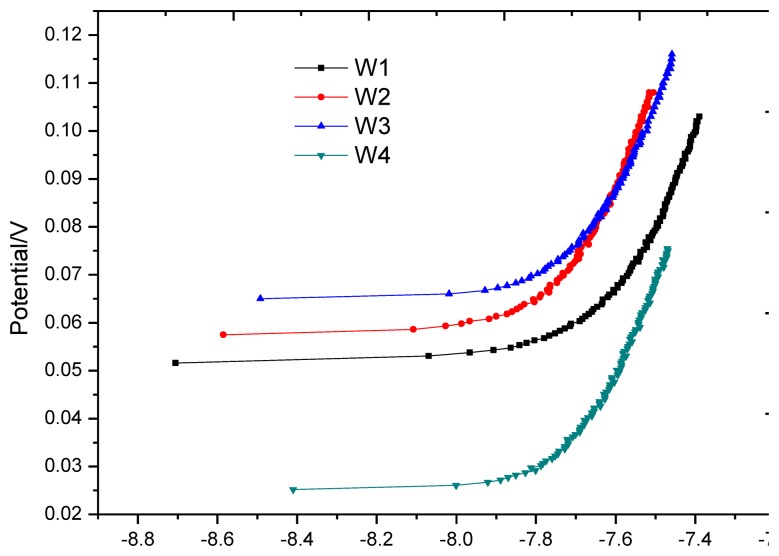
Anodic polarization curve for each monitoring point after first drying process.

After the first air-drying cycle, the anodic polarization curve test was applied at each monitoring point again, as shown in Figure 13. In contrast with Figure 10, the shape of all polarization curves in Figure 13 has the typical characteristics of anodic polarization, showing that oxygen supply returns to equilibrium. Table 5 shows that the equilibrium potential at monitoring point is in a sharp positive shift and the offset is greater than 100 mV; anodic polarization current changes from negative value to positive value, both significantly less than the corrosion threshold 0.754 *u*A, showing that all monitoring points are in passivation.

Compared with the test methods used for macro currents, every time the polarization test ends, a high-sensitivity and zero-resistance ammeter (range: 0–10 μA, accuracy: 0.5 μA) is used to measure the macro current between the auxiliary electrode and working electrode at half an hour intervals, and then the value is recorded after stable connection. Before the corrosion of the monitoring point W1 is determined, the test is performed the next day after each cycle soaking ends. When the corrosion of the monitoring point W1 is determined, the sample is placed in a box with a constant temperature of 30 °C and a constant humidity of 40% and then an accelerated air drying is performed for three days, with a test each day.

**Figure 14.**
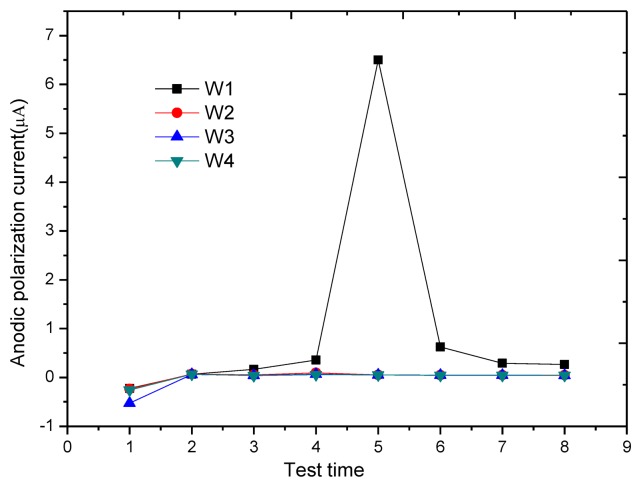
Variation of anodic polarization current for each monitoring point.

**Figure 15.**
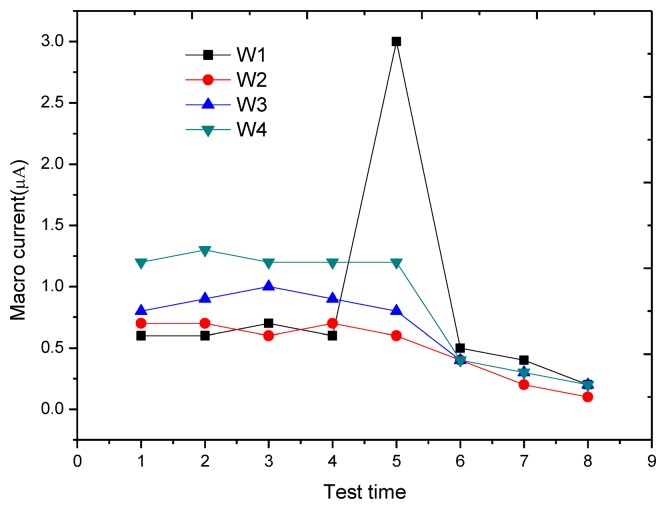
Variation of macro current for each monitoring point.

Figure 14 shows the change of anodic polarization current *I_APC_* at each monitoring point. At the monitoring point W1, starting from the third test, the anodic polarization current begins to increase gradually (from 60 nA in stable passivation state to 268 nA), showing that the passivation membrane of reinforcement gradually becomes instable. In the fifth test, the anodic polarization current increases sharply to 6.56 μA, showing that reinforcement begins to rust. During the three-day air drying after corrosion, the anodic polarization current changes significantly. On the first day of air drying, the anodic polarization current decreases to 628 nA, a value below the critical corrosion anodic polarization current of 754 nA, and stabilizes at around 260 nA during the last two days. This is because air drying accelerates the moisture evaporation on the block surface so that the concrete resistance increases and the corrosion current at W1 decreases sharply. One point to mention is that in the three-day air drying, although the anodic polarization current is lower than the corrosion threshold, it is still significantly greater than the anodic polarization current in stable passivation. In contrast with the results of the macro current test, the macro current at W1 also increases sharply to 3.1 μA in the fifth test, as shown in Figure 15, indicating that the reinforcement began to rust and the result is consistent with the determined result of the anodic polarization current, but in previous tests, no signs of reinforcement corrosion appear, and the test value is even lower than those at other monitoring points. In addition, during the three-day air drying, the macro currents at all monitoring points reduce to a similar value, making it hard to distinguish the rusted monitoring point W1.

The above analysis shows that compared with the macro current criterion, the anodic polarization current method can be used to both determine whether the monitoring point rusts or not, and characterize the whole development of corrosion; after corrosion, even if the monitoring point is in the dry state, it remains significantly different from the stable passivation state.

### 3.4. Impact of Ohmic Drop

During electrochemical tests, if the concrete resistance is bigger, the potential loss caused by polarization current flows can't be ignored [21]. On the premise that no ohm compensation is used, the resistance $R_{P}^{\prime }$ obtained with linear polarization method actually contains the concrete cover resistance *R_c_*, *i.e.*, the real polarization resistance should be:

(8) $$R_{P}=R_{P}^{\prime }-R_{C}$$

Equation (8) indicates that a bigger *R_c_* will make a bigger $R_{P}^{\prime }$, while the measured corrosion current will be smaller. In order to measure accurate corrosion current, the impact of ohmic drop should be reduced to a minimum level.

**Figure 16.**
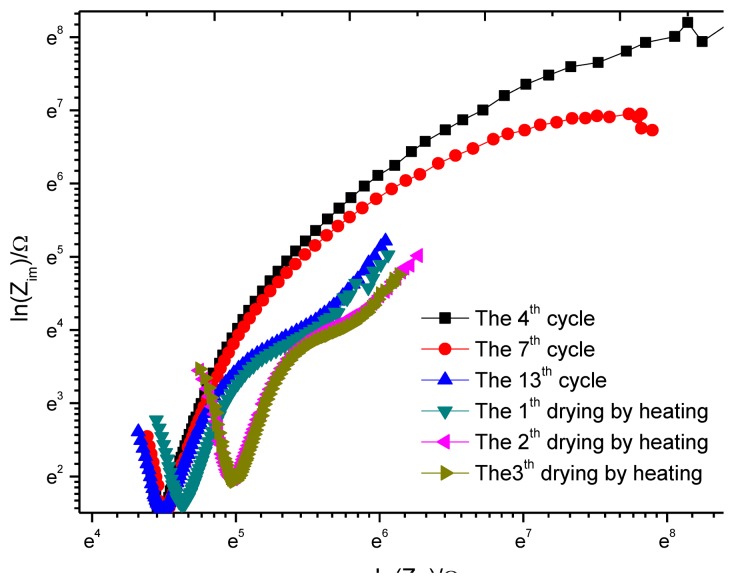
Nyquist plots of monitoring point W1.

In sensor design, to reduce the impact of ohmic drop, the distance between the reference electrode and working electrode will be limited at 3 mm. To study the impact of ohmic drop, after the 13th dry-wet circulation ends, the sample is placed in a box with a constant temperature of 30 °C and a constant humidity of 40% and then an accelerated air drying is performed for three days, with a test each day. The result is shown in Figure 16. For the purpose of comparison, the impedance spectrum data of the 4th, 7th and 13th circulation is added. Considering the impedance change is very big, the coordinates are changed to logarithmic coordinates. In the figure, the corresponding abscissa value at the knee point of curve V is the resistance of concrete cover. As the drying times increase, the knee point moves to the right, showing that the resistance of concrete cover increases gradually. After the third drying, the resistance no longer increases and stabilizes.

To analyze the influence of concrete resistance on the test result, the equivalent circuit as shown in Figure 7 is used for curve fitting. The results are shown in Table 6. In Figure 7, *R**_s_* represents the solution resistance, *R_c_* represents the concrete resistance, *R_r_* represents the rust layer at the monitoring point W1, and *R_p_* represents the polarization resistance. If set *σ*= *(R_c_*+*R_p_)*/ *R_p_*, and σ is defined as the impact factor of ohmic drop, then:

(9) $$I_{\text{corr}}^{\prime }=I_{\text{corr}}\cdot \sigma$$

In the equation, *I_corr_* is the test value of corrosion current, $I_{\text{corr}}^{\prime }$ is the corrosion current that eliminates the impact of ohmic drop. Table 6 shows that during the passivation of reinforcement, the impact of the ohmic drop can be neglected. As the corrosion develops, *R_p_* gradually decreases, and the impact of the ohmic drop begins to increase. As the drying degree increases, the concrete resistance *R*_c_ increases gradually, but *R_p_* also increases, and the impact of ohmic drop increases slightly. In the whole process, σ is less than 1.0642, indicating that the ohmic drop has a very small impact on the sensor test results.

### 3.5 The Relationship Between Anodic Polarization Current and Corrosion Current

**Figure 17.**
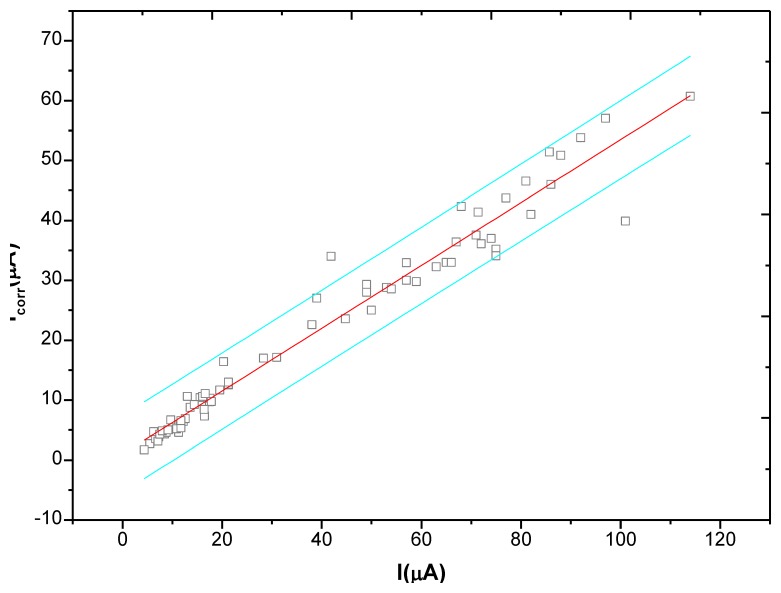
Relationship between corrosion current and anodic polarization current.

In the monitoring process, after the anodic polarization current test ends, the linear polarization method was used again, at an interval of 20 minutes, to test the corrosion current *I_corr_* at the monitoring point. The test results are shown in Figure 17. If can be clearly found in the figure that there is an obvious linear relationship between the anodic polarization current *I_APC_* and the corrosion current *I_corr_*. After regression, we get Equation (9). Therefore, in the anodic polarization current method, by simply recording the anodic polarization current *I_APC_* at the end of polarization, the corrosion current *I_corr_* at monitoring point can be obtained quickly without the polarization curve fitting analysis, making the test simple and rapid. Furthermore, the critical corrosion current density to represent rebar starting corrosion is about 0.1∼0.2 uA/cm^2^ [22], according to Equation (9), the critical anodic polarization current density *i_cr_* can be obtained at about 0.15∼0.3 uA/cm^2^:

(9) $$I_{\text{corr}}=0.67I_{\text{APC}}(\mu A)$$

## 4. Conclusions

This paper presents a new corrosion sensor design based on a three-electrode electrochemical test architecture, and proposes an anodic polarization current method to determine the corrosion of reinforcements based on electrochemical polarization. The main conclusions are as follows:

(1) The working electrode and the reference electrode are symmetrically distributed on both sides of the auxiliary electrode, so that when the auxiliary electrode polarizes the working electrode on one side, the polarization current field created will not have a polarization effect on the working electrode on the other side.
(2) The clear distance between reference electrode and working electrode is only 3 mm. The ohmic drop of concrete resistance has a very small impact on the test result.
(3) The features of anodic polarization curve can effectively characterize the oxygen supply at the monitoring points in concrete. Due to the negative shift of the equilibrium potential at the monitoring point caused by oxygen deficit, the anodic polarization curve has cathodic polarization characteristics, but the corrosion condition at each point can still be determined by the anodic polarization current I_APC_.
(4) Compared with the macro current criterion, the anodic polarization current method can be used to determine both the corrosion status at the monitoring point and characterize the whole development of passivation membrane. After the corrosion occurs at the monitoring points, even if the point is in the dry state, it remains significantly different from stable passivation.
(5) There is an obvious linear relationship between the anodic polarization current *I_APC_* and the corrosion current *I_corr_*.

## Figures and Tables

**Table 1.** Mixture proportions of the concrete block.

**Water**	**Cement**	**Sand**	**Aggregate**
195	433	569	1156

**Table 2.** Potential of monitoring points.

**Monitoring Point**	**W1**	**W2**	**W3**	**W4**
*E_oc_ v.s.* Ti	−85 mV	−93 mV	−98 mV	−157 mV
*E_oc_ v.s.* SCE	−390 mV	−405 mV	−420 mV	−508 mV

**Table 3.** Anodic polarization current of monitoring points.

**Monitoring Point**	**W1**	**W2**	**W3**	**W4**
*I_APC_*	−5.311 nA	2.529 nA	−45 nA	−875 nA

**Table 4.** Anodic polarization current of monitoring points after drying for one month.

**Monitoring Point**	**W1**	**W2**	**W3**	**W4**	
*I_APC_*	64.4 nA	60.15 nA	67.46 nA	62.71nA	

**Table 5.** Anodic polarization current and potential at monitoring points.

**Monitoring Point**	**W1**	**W2**	**W3**	**W4**
*E_oc_* after wetting	−60 mV	−75 mV	−95 mV	−132 mV
*E_oc_* after drying	53 mV	58 mV	66 mV	26 mV
*I_APC_* after wetting	−226 nA	−235 nA	−528 nA	−259 nA
*I_APC_* after drying	40.78 nA	31.26 nA	34.84 nA	33.88 nA

**Table 6.** Curve fitting results for Nyquist plots of monitoring point W1.

	**4th Cycle**	**7th Cycle**	**13th Cycle**	**1th Drying by Heating**	**2th Drying by Heating**	**3th Drying by Heating**
*R_c_*	81.4	80.6	81.3	88.5	123.1	130.5
*R_p_*	9989	3308	1408	1514	1918	2043
*(R_c_* + *R_p_)*/*R_c_*	1.0082	1.0244	1.0578	1.0585	1.0642	1.0619
